# Data set on the influence of members of a couple on family vacation decision-making

**DOI:** 10.1016/j.dib.2019.104233

**Published:** 2019-07-26

**Authors:** María-Mercedes Rojas-de-Gracia, Pilar Alarcón-Urbistondo, Ana-María Casado-Molina

**Affiliations:** Department of Economics and Business Administration, University of Málaga, Spain

**Keywords:** Couple roles, Family decision-making, Influence of the couple, Family vacations, Perceived partner influence, Response agreement, Dyadic consensus

## Abstract

This article describes a database on the perceptions of members of a couple regarding the influence exerted in three stages (initiation, search, and final decision) and seven sub-decisions (destination, accommodation, transport, budget, date, activities, restaurants) in the family vacation decision-making process. We obtained responses from 375 couples, whose members each completed the questionnaire individually, which also enables researchers to obtain information about the consensus between the perceptions of both regarding said influence. To complement this information, we also included sociodemographic and travel behavior variables for the couples analyzed. The data are publicly available at https://github.com/mmrojasgracia/Data-in-Brief_Influence. For interpretation and discussion, please see the original article entitled “Is asking only one member of a couple sufficient to determine who influences tourism decisions?” (Rojas-de-Gracia et al., 2019) (Rojas-de-Gracia et al., 2019).

**Table:** Specifications table

Subject area	*Marketing, Tourism, Sociology*
More specific subject area	*Tourism Consumer Behavior, Sociology of the Family*
Type of data	*SAV file*
How data was acquired	*Self-administered questionnaires distributed in schools to adolescent students who in turn delivered them to their parents*
Data format	*Raw*
Experimental factors	*Questionnaires were excluded if they were completed by only one member of the couple or presented discrepancies in aspects in which both questionnaires should have coincided*
Experimental features	*In the questionnaire dyads, which fathers and mothers were asked to complete individually, we asked participants about their perceptions of the influence exerted on different aspects and sub-decisions of their last family vacation*
Data source location	*Andalucia (Spain)*
Data accessibility	*The data and the questionnaire are publicly available at*https://github.com/mmrojasgracia/Data-in-Brief_Influence
Related research article	*M.M. Rojas-de-Gracia, P. Alarcón-Urbistondo, A.M. Casado-Molina* *Is asking only one member of a couple sufficient to determine who influences tourism decisions?* *Journal of Destination Marketing & Management**https://doi.org/10.1016/j.jdmm.2019.03.005*

**Table:** 

**Value of the data** • The data can serve as a model for researchers interested in studying the influence of members of a couple in family decision-making, and consensus between their perceptions of this, aspects about which much remains to be elucidated. • In view of the difficulty of obtaining two responses per household, the data collected in this study can be used by future researchers who wish to add data on new couples, thus achieving larger samples. • These data can be used to conduct comparisons of samples of couples in different countries, in order to determine the existence of differences in the roles played by couples in tourism decisions and of consensus in their perceptions, according to nationality. • Given that the data include sociodemographic characteristics of couples traveling as a family, future researchers could use these data to perform comparative studies, in order to detect sociodemographic similarities and differences of this type of couples according to different nationalities. • Data on the travel behavior characteristics of couples traveling as a family can be used to conduct comparative studies of the patterns of travel behavior of other family structures, such as single couples, single-parent families or extended families.

## 1 Data

The data described in this article contain responses about the perceptions of each member of a couple regarding who was the most influential member in various aspects of family vacation decision-making. These aspects refer to the three most frequently studied stages of tourist decision-making: Initiation, information search, and final decision [2], [3], [4], and seven sub-decisions that have received considerable research attention: Destination, accommodation, transport, budget, date, activities, and restaurants [5], [6], [7], [8]. Table 1 shows the variables collected in the data regarding the influence exerted by each member of the couple, as well as their respective categories. Since men and women responded separately to identical questionnaires, the data include two responses for each of the aspects analyzed: the perceptions of men and of women. In addition, Table 2 presents the variables and categories relating to the sociodemographic and travel behavior characteristics of the couples. Note that each member was asked about their age, employment status and educational level, so each one answered these questions in their corresponding questionnaire. A summary of the data obtained can be found in the study [1].

**Table 1:** Data on influence of members of a couple on family vacation decision-making.

Type of variable	Variable	Categories
Stages/sub-decisions	Initiator according to man/woman	Woman
	Searcher according to man/woman	Joint
	Final decision-maker according to man/woman	Man
	Who chose the destination according to man/woman?	No one
	Who chose the accommodation according to man/woman?	Others
	Who chose the means of transport according to man/woman?	999^a^
	Who chose the budget to spend according to man/woman?	
	Who chose the vacation date according to man/woman?	
	Who chose the activities according to man/woman?	
	Who chose the restaurants according to man/woman?	

a The 999 code corresponds to missing data.

**Table 2:** Data on sociodemographic and travel behavior characteristics of couples.

Type of variable	Variable	Categories
Sociodemographic	Family type	Traditional family
		Restructured family
	Type of union	Married in church
		Married in a registry office
		Not married
	Age of man	[Numerical variable]
	Age of woman	[Numerical variable]
	Employment status of man	Unemployed man
		Employed man
	Employment status of woman	Unemployed woman
		Employed woman
	Educational level of man	No schooling
		Elementary school
		High school
		College education
	Educational level of woman	No schooling
		Elementary school
		High school
		College education
	Length of time living together	Less than 20 years
		20 years or more
Travel behavior	Purpose	Exclusively for leisure
		Visit family and friends
		Others
	Type of destination	Domestic
		International
	Frequency of vacations	Sporadic
		Every two or three years
		At least every year
	Travel group composition	Couple and children
		Couple, children, and others
	Travel organization	Independent
		Agency

Note: For all variables, the code 999 corresponds to missing data.

Given that collecting two responses per household makes it impossible in practice to perform random sampling, it was necessary to study the representativeness of the sample. Since numerous studies have used the variable of educational level as a determinant of the different structures of vacation decisions [6], [9], [10], [11], [12], [13], [14], [15], [16] and given that it is objectively measurable and more stable than other variables as the employment status of partners, we used it to compare study population and study sample data, as shown in Table 3.

**Table 3:** Comparison of educational level between families in the study population and both samples.

Type of individuals	Educational level	No. study subjects (%)
Study population^a^	No schooling	b
	Elementary school	782,019 (36.4%)
	High school	813,476 (37.9%)
	College education	551,464 (25.7%)
Men (study sample)	No schooling	9 (2.4%)
	Elementary school	129 (34.5%)
	High school	160 (42.8%)
	College education	76 (20.3%)
Women (study sample)	No schooling	6 (1.6%)
	Elementary school	103 (27.6%)
	High school	168 (45.0%)
	College education	96 (25.7%)

a *Source*: TOURSPAIN (2015).

b Blank figures correspond to an insufficient sample (fewer than 100 records).

## 2 Experimental design, materials, and methods

Given the heterogeneity of couples, these data were collected from one particular type: Heterosexual couples who were living together and had at least one child in common aged between 10 and 18 years old who would have accompanied them on the vacation. In order to prompt accurate recollections, participants were asked to think about their last family vacation. In accordance with [17], we defined “vacation” as a trip outside the family home lasting at least three days and whose main purpose was leisure. Children were included because this type of family constitutes a very important consumer for the tourism industry, and the couples’ decisions affect their children as well as themselves.

To obtain responses from men and women in the same couple, questionnaires were distributed from January to April of 2016 to pupils attending several public and private schools in different areas of coastal and inland Andalusia, in the south of Spain, and they were asked to pass them on to their parents. These questionnaires were preceded by a letter stressing the importance of both parents filling them in separately. They were also asked to agree on the vacation on which they would base their responses.

A total of 1,200 questionnaire dyads were distributed, and a final total of 375 dyads suitable for analysis were obtained. There were three fundamental reasons for excluding questionnaires. The first was that some had only been completed by one of the partners, mainly because the parents did not live together. The second was the existence of inconsistencies in fields that both members of the couple had to complete and which ought to match, for example, the length of time they had been living together or the composition of the travel group. However, there were very few inconsistencies given that this concerned objective data. The third reason was simply that some questionnaires were not returned to the tutors. Non-response is a frequent problem, especially when requesting two responses per household, and one that is difficult to remedy. To overcome the problems entailed in convenience samples typical of this type of study and given the complexity of obtaining two responses per household, interviews were held with the students’ tutors to clarify possible reasons for non-response. These stated that non-response was due to disinterest on the part of parents and/or children. Therefore, it did not indicate any kind of systematic bias, which would be the most problematic type of error.

In order to analyze sample representativeness of the study population, we used data published by the Spanish Tourism Institute (Tourspain). This survey examined the sociodemographic profile of households resident in Spain, taking as influential variables those related to household composition, the employment status of members, educational level of the head of the family and the availability of a second home [18]. An email was sent to Tourspain asking them to indicate how the proportion of families traveling with children aged 10 to 18 was distributed according to educational level. Using the Mann-Whitney U test, we compared the proportions of individuals according to Spanish Tourism Institute data and study sample data. No differences were found between the study population and the sample of men (p = 0.9276) or between the study population and the sample of women (p = 0.3831). Therefore, our sample can be considered representative of the study population.
