# Supplementary material for: Data set on the influence of members of a couple on family vacation decision-making
Source: Data Brief. 2019 Jul 26;25:104233. doi: 10.1016/j.dib.2019.104233 (PMC6702380; doi:10.1016/j.dib.2019.104233)
Supplement: Supplementary file 1 [file mmc1.docx]

**QUESTIONNAIRE**

**INSTRUCTIONS**: *This questionnaire consists of two parts, one for each partner. It is very important that each member fills in his/her part separately. Previously, you should agree on the last vacations that all three went together, although other people were with you, as long as at least one son/daughter were between 10 and 18 years of age when you were on that vacation. If necessary, reach an agreement among yourselves so that all the answers refer to the same vacations.*

*Besides, on that vacation you must stay at least THREE DAYS AWAY FROM HOME with the main objective of leisure.* *If you don't meet these requirements, there is no need to fill in the survey. Otherwise, please, complete it and, when you finish, make sure that the child delivers the three questionnaires to the person who gave them.*

*All data collected is completely anonymous.*

1. Gender:

Male

Female

1. How old are you?
2. Do you or your partner have a child from previous partners?

No, we are a traditional family

Yes, we are a restructurated family

1. Your marital status with your current partner is:

Married in church

Married by civil law

Not married

1. Did you have a job when you went on that vacation?:

Yes

No

1. Your level of education is:

None

Elementary school

High school

College education

1. How long have you been living with your current partner?:

Less than 20 years

20 years or more

1. What kind of destination you were?:

Domestic (within the country)

International (abroad)

Please, say exactly where did you go:

1. Why did you go to that destination?:

Exclusively for leisure

Visit family and friends

Others (say what):

1. How often do you go on vacation?:

At least once a year

Every two or three years

Sporadic

1. Besides the couple and the children, did anyone else travel with you on that vacation?:

Yes (say who):_________________________________________________________

No

1. How did you organize the vacation?

Through an agency

Independently

1. Who proposed to go to that vacation? Please, mark just one option:

Mainly from me

Mainly from my partner

From my partner and me in equal proportion

Others

No one

1. Who searched the holiday information, for example, about the destination or hotels (on the Internet, catalogs, asking someone...)? Please, mark just one option:

Mainly from me

Mainly from my partner

From my partner and me in equal proportion

Others

No one

1. Who was the person who took a definitive decision and finally decided that you were going on vacation? Please, mark just one option:

Mainly from me

Mainly from my partner

From my partner and me in equal proportion

Others

No one

1. Who do you think was most influential in the following decisions on your last vacation? Please, mark just one option:
   1. Destination:

I more than my partner

I less than my partner

My partner and I in equal proportion

Others

No one

- 1. Accommodation:

I more than my partner

I less than my partner

My partner and I in equal proportion

Others

No one

- 1. Means of transport:

I more than my partner

I less than my partner

My partner and I in equal proportion

Others

No one

- 1. Budget:

I more than my partner

I less than my partner

My partner and I in equal proportion

Others

No one

- 1. Date:

I more than my partner

I less than my partner

My partner and I in equal proportion

Others

No one

- 1. Activities and tours during the vacation:

I more than my partner

I less than my partner

My partner and I in equal proportion

Others

No one

- 1. Restaurants to eat:

I more than my partner

I less than my partner

My partner and I in equal proportion

Others

No one

THANK YOU FOR YOUR COOPERATION

Please, return the questionnaire to your child
